# Tiamulin inhibits breast cancer growth and pulmonary metastasis by decreasing the activity of CD73

**DOI:** 10.1186/s12885-017-3250-4

**Published:** 2017-04-11

**Authors:** Xu Yang, Shimin Pei, Huanan Wang, Yipeng Jin, Fang Yu, Bin Zhou, Hong Zhang, Di Zhang, Degui Lin

**Affiliations:** 1 The Clinical Department, College of Veterinary Medicine, China Agricultural University, Beijing, 100193 China; 2 Department of Veterinary Medicine, College of Animal Sciences, Zhejiang University, Hangzhou, 310058 China

**Keywords:** Breast cancer, CD73, Thf, Metastasis, Angiogenesis

## Abstract

**Background:**

Metastasis is the leading cause of death in breast cancer patients. CD73, also known as ecto-5′-nucleotidase, plays a critical role in cancer development including metastasis. The existing researches indicate that overexpression of CD73 promotes growth and metastasis of breast cancer. Therefore, CD73 inhibitor can offer a promising treatment for breast cancer. Here, we determined whether tiamulin, which was found to inhibit CD73, was able to suppress breast cancer development and explored the related mechanisms.

**Methods:**

We firstly measured the effect of tiamulin hydrogen fumarate (THF) on CD73 using high performance liquid chromatography (HPLC). Then, we investigated cell proliferation, migration and invasion in MDA-MB-231 human breast cancer cell line and 4 T1 mouse breast cancer cell line treated with THF by migration assay, invasion assay and activity assay. Besides, we examined the effect of THF on syngeneic mammary tumors of mice by immunohistochemistry.

**Results:**

Our data demonstrated that THF inhibited CD73 by decreasing the activity instead of the expression of CD73. In vitro, THF inhibited the proliferation, migration and invasion of MDA-MB-231 and 4 T1 cells by suppressing CD73 activity. In vivo, animal experiments showed that THF treatment resulted in significant reduction in syngeneic tumor growth, microvascular density and lung metastasis rate.

**Conclusions:**

Our results indicate that THF inhibits growth and metastasis of breast cancer by blocking the activity of CD73, which may offer a promising treatment for breast cancer therapy.

**Electronic supplementary material:**

The online version of this article (doi:10.1186/s12885-017-3250-4) contains supplementary material, which is available to authorized users.

## Background

CD73, also known as ecto-5′-nucleotidase, encoded by NT5E gene, is a widely distributed cell-surface protein anchored on the plasma membrane via a glycosyl phosphatidylinositol linkage. CD73 functions via both enzymatic and nonenzymatic activities in cells [1]. As a nucleotidase, CD73 catalyzes the conversion of AMP to adenosine (Ado), a crucial step in the pathway of Ado generation. Ado binds and activates four Ado receptors: A1, A2A, A2B and A3 receptors, exerting their various biological effects [2]. Besides its essential physiological functions, Ado plays an important role in cancer development. The activities of Ado in cancer include growth promotion, angiogenesis, immunosuppression, neointimal hyperplasia and anti-inflammation [1, 3–5]. The activities of Ado in cancer were mainly achieved by activating Ado receptors. Via activation of A1 receptor, Ado decreases p27 expression to up-regulate CDK4 activity, which results in cell proliferation [6]. Overexpression of A1 receptor has been found in various breast cancer cell lines and breast tumors [6]. Activation of A2A receptors has been proven to directly promote tumor cell survival. Indeed, Etique et al. showed that the activation of A2A receptor significantly increased the proliferation of breast cancer cell [7]. Ahmad et al. also reported that the activation of A2A receptor remarkably enhanced tumor angiogenesis [8]. In addition, the A2A receptor is a potent suppressor of endogenous tumor immunity. Ohta et al. revealed that A2A receptor inhibition enhanced existing antitumor immune responses [9]. Ado also promotes cell proliferation, dissemination and angiogenesis by activating A2B receptor. The activation of the A2B receptor has been shown to enhance cell proliferation in prostate cancer cell lines [10]. The blockade of A2B receptor slows growth of bladder and breast tumors. Stagg et al. demonstrated the ability of adenosine to increase tumor cell migration and metastasis via binding to A2B receptor [11]. Besides, the activation of A2B receptor induces the production of IL-4, IL-8, IL-13 and vascular endothelial growth factor [12]. Adenosine also regulates tumor cell proliferation through A3 receptors [13–15]. As a key enzyme in the generation of Ado, CD73 plays a critical role in cancer development. In addition to its function elicited by the production of Ado, CD73 is involved in the promotion of cancer directly. CD73 could mediate cancer invasive and metastatic properties by regulating cell interaction with extracellular matrix (ECM) components, like laminin and fibronectin [16, 17]. Actually, both enzymatic and nonenzymatic functions of CD73 are associated with cancer progression and not completely independent of each other [18]. Overexpression of CD73 could facilitate malignant behaviors of carcinomas, such as growth, adhesion, migration, invasion and metastasis [19]. Recently, CD73 overexpression has been reported in many types of human and mouse cancers, including breast cancer [11, 19], gastric cancer [20], pancreatic cancer [21], colorectal cancer [22, 23], prostate cancer [24] and thyroid cancers [25]. The vital role of CD73 in cancer development has been highlighted.

Breast cancer is the second most common cancer worldwide and, by far the most frequent cancer in women, comprising about a quarter of all female cancers, as well as a leading cause of cancer death in women [26]. In breast cancer, tissue invasion and metastasis is the most happened outcome resulting in lethal. It is very meaningful to understand the molecular mechanisms involved in breast cancer and find more effective treatment target site [27]. In breast cancer, CD73 overexpression is associated with a highly aggressive cancer phenotype, tumor promotion, and drug resistance [11]. Inhibition of CD73 can suppress cell growth, invasion in breast cancer and reduce tumor growth and metastasis [11, 28]. Targeted cancer therapy is supposed to be more effective than conventional treatments and less harmful to normal cells. Anti-CD73 targeted therapy may offer a promising treatment for breast cancer containing high level of CD73.

In our study, we found tiamulin, used in the form of tiamulin hydrogen fumarate (THF), could inhibit the activity of CD73. Tiamulin is a derivative of the tricyclic diterpenoid compound pleuromutilin, a product isolated from the *Clitopilus scyphoides* [29]. Pleuromutilins inhibit targeted protein synthesis by interfering with the large subunit of the bacterial ribosome. Tiamulin, as an antibiotic, is mainly used to treat dysentery and respiratory diseases in veterinary medicine [29]. Ronald N. Jones etc. also reported that tiamulin remained antimicrobial activity against anaerobes, intestinal spirochetes, and many common isolates of *staphylococci* and *streptococci* from animal and human [30]. Tiamulin is widely used in the form of THF. In this paper, based on its function of inhibiting CD73, THF was firstly studied as a targeted anti-tumor medicine in breast cancer, which was never reported before in the light of our knowledge.

## Methods

### CD73 activity inhibition in vitro

In this assay, all reagents including tiamulin hydrogen fumarate (THF), Adenosine monophosphate (AMP) and Adenosine (Ado) were purchased from Sigma-Aldrich (St Louis, MO, USA), except Recombinant Human 5′-Nucleotidase/CD73 Protein (rhCD73) (R&D Systems, MN, USA). CD73 activity was analyzed by measuring the conversation of AMP to Ado with high performance liquid chromatography (HPLC) system (Agilent Technologies Inc., CA, USA). Briefly, rhCD73 (100 ng/mL) was prepared in assay buffer (25 mM Tris, 5 mM MgCl2, pH 7.5). Then, assay buffer alone (control) or with THF (5, 10 or 20 μM) was added. After 10 min, AMP (100 μM) was added for an additional 10 min. The production of Ado was detected by HPLC at 254 nm, and CD73 activity was expressed as Ado production per mg protein in 10 min. As well as THF, α, β-Methylene adenosine-5′-disphosphate (APCP, a specific inhibitor of CD73) (10 μM) or tylosin (an antibiotic similar to THF in antibiotic spectrum) (10 μM) was used in this assay as positive or negative control.

### Cell culture

In this study, the human breast cancer line MDA-MB-231 and the mouse breast cancer line 4 T1 purchased from American Type Culture Collection (ATCC, Manassas, VA, USA) were used. MDA-MB-231 was cultured in DMEM medium (gibco, life technologies, NY, USA) supplemented with 10% fetal bovine serum (FBS) (gibco, life technologies) and penicillin-streptomycin (100 units/mL). 4 T1 was cultured in RPMI 1640 medium (gibco, life technologies) supplemented with 10% FBS and penicillin-streptomycin (100 units/mL). Both cell lines were cultured in a humidified atmosphere of 5% CO_2_ at 37 °C.

### Cell proliferation evaluation

Cell viability was analyzed using a Cell Counting Kit-8 (CCK-8) (Beyotime, Jiangsu, China). MDA-MB-231 or 4 T1 cells were seeded in 96-well plates at 2 × 10^4^ cells per well and incubated overnight before attached. Then, cells were treated without (control group) or with different concentrations of THF (0, 6.25, 12.5, 25, 50 or 100 μg/ml). After 0, 12, 24, 36 or 48 h, cell viability was assessed with CCK-8 according to the manufacturer’s instructions. To determine the cell viability, the optical density (OD) values were measured at 450 nm using a microplate reader (Bio-Rad, CA, USA). Cell proliferation was determined as a percentage of the control wells.

In the colony formation assay, MDA-MB-231 or 4 T1 cells in single-cell suspension with solvent alone (control) or different concentrations of THF (12.5 or 25 μg/mL) were seeded in 12-well plates at a density of 2000 cells per well. After 24 h treatment, the cell debris and unattached cells were washed out and fresh medium without THF were added into each well. After 7 days of incubation, colonies were fixed with methanol and stained with 0.1% crystal violet (Solarbio, Beijing, China). Visible colonies (>50 cells/colony) in the wells were manually counted and compared.

### CD73 activity in cells

The CD73 activity assay was performed as described by Sherene Loi et al. [31]. MDA-MB-231 or 4 T1 was plated at 10^4^ cells per well in a 96-well plate and cultured in complete medium. After 24 h, culture medium was removed and cells were washed twice with prewarmed phosphate-free assay buffer (2 mM MgCl2, 125 mM NaCl, 1 mM KCl, 10 mM glucose, 10 mM Hepes pH 7.2, diluted in ddH2O). THF (12.5 or 25 μg/mL) diluted in assay buffer or buffer alone (control) was added to cells and incubated at 37 °C for 10 min. AMP (250 μM) diluted in assay buffer or buffer alone was then added and incubated at 37 °C for 1 min. Phosphate concentrations resulting from AMP hydrolysis by CD73 were measured using the malachite green phosphate detection kit (R&D Systems, MN, USA) following the manufacturer’s instructions. For further confirmation, THF, as well as APCP or tylosin (25 μg/mL), was tested again in this assay.

### Western-blot

Western-blot analysis was used to detect the expression of CD73 protein in MDA-MB-231 and 4 T1 cells. Cells were randomly plated in a 6-well plate and treated with THF (12.5 or 25 μg/ml) or solvent alone (control). After 36 h, cells were collected and lysed, and then proteins were isolated and quantified using the BCA protein assay kit (Beyotime, Jiangsu, China). Twenty micrograms of each sample were separated by 10% SDS-PAGE, transferred to PVDF membranes (MercK Millipore, MA, USA). The membranes were blocked in 5% milk for 2 h and incubated with primary antibody against CD73 (Abcam, Cambridge, USA) or GAPDH (Santa Cruz Biotechnology, CA, USA) (as loading control) overnight, followed by secondary antibodies for 2 h, and then detected by enhanced chemiluminescence detection reagent (Millipore Corporation, MA, USA).

### Cell migration and invasion assays

In the cell migration and invasion assays, a 24-well transwell plate (lower chamber) and transwell inserts (upper chamber) with 8 μm pore size polycarbonate membrane (Corning Incorporated, NY, USA) were used. In the migration assay, MDA-MB-231 or 4 T1 cells (5 × 10^4^/mL) in 100 μL serum-free media with solvent (control) or different concentrations THF (12.5 or 25 μg/mL) were added to the upper chamber, meanwhile the lower chamber was filled with 600 μL media containing 10% FBS. After 36 h of incubation, tumor cells in the upper chamber were removed carefully. The cells that migrated through the membrane to the lower surface were fixed with methanol, stained with 0.1% crystal violet. Cell numbers were counted as the means ± SD of cells in five separate views based on three independent experiments. In the invasion assay, 5 × 10^3^ cells in 100 μL serum-free media were added to the upper chamber, which was precoated with 30 μg Matrigel Matrix (Corning Incorporated). The lower chamber was filled with 600 μL media containing 10% FBS. After 36 h of incubation, Matrigel Matrix and cells in the upper chamber were removed carefully, and then cells adhering to the lower surface of the membrane were counted. To investigate the relevance of the inhibition effects of THF on CD73 activity to tumor migration and invasion, exogenous Ado was added to THF treatment groups in parallel experiments.

### Animal experiment

Four-week old female BABL/c mice used in this study were purchased from Vital River (Beijing, China). After a week of acclimatization, 4 T1 cells (1.5 × 10^6^) suspended in 100 μL PBS or PBS alone (normal control) were injected subcutaneously into the right flank of each mouse. After 5 days, tumor bearing mice were randomized into positive control, low-dosage and high-dosage groups (6 mice in each group). By intragastric administration once per day, the mice in normal and positive control groups were treated with solvent alone; those in low-dosage and high-dosage groups were treated with low-dose (80 mg/kg body weight) and high-dose (160 mg/kg body weight) THF, respectively. Meanwhile, body weights and tumor volumes were regularly monitored during the course of treatment. Tumor volumes were calculated as height × width 2/2. After 21 days of treatment, mice were sacrificed for collections of syngeneic tumors and lung tissues. All animal procedures were performed with the approval of the Institutional Animal Care and Use Committee of China Agricultural University.

### Immunohistochemistry

After removed from the mice, the syngeneic tumors and lung tissues were fixed with neutral-buffer formalin, embedded in paraffin wax and sectioned serially at 4 μm. Immunohistochemistry (IHC) staining was performed by using antibodies for the proliferation marker protein Ki-67 (anti-Ki67) (mouse monoclonal, ZSGB-BIO, China), cluster of differentiation 31(anti-CD31) (rabbit polyclonal, Bioss, China). After incubated with biotinylated secondary anti-mouse or anti-rabbit antibody IgG (ZSGB-BIO, Beijing, China), tissue sections were firstly developed with 3, 3′-diaminobenzidine (DAB) and then counterstained with hematoxylin. After dehydrated and mounted, the staining tissues were observed under a microscope and analyzed by Image-pro-plus (IPP) software (Media Cybernetics, Washington, USA).

### Statistical analysis

All experiments were performed in three independent experiments and results are expressed as mean ± standard deviation (SD). The results are analyzed using SPSS 20 software (Statistical Product and Service Solutions, Chicago, USA). The analysis of one-way ANOVA or Student’s t-test (when only 2 groups) was used to identify the differences between groups. *P* < 0.05 was regarded as statistically significant.

## Results

### CD73 activity was significantly decreased by THF

**Fig. 1:**
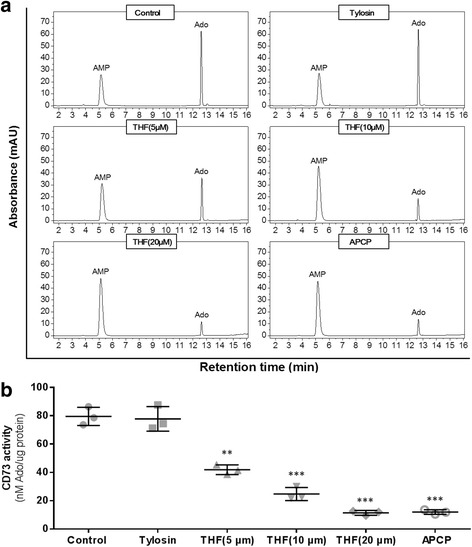
THF decreased the activity of CD73. **a** Representative high performance liquid chromatography (HPLC) of AMP and Ado in control and THF treatment group. The absorbance was detected by UV at 254 nm. **b** CD73 enzyme activity was expressed as Ado productions per mg protein in 10 min. Data represent the mean ± S.D. of three independent experiments. Peak identities were confirmed by comparing the UV spectra and retention times of samples with standard compounds. Ado productions were obtained by comparing the peak area of samples with standard. (**P < 0.01, ***P < 0.001 vs. control)

To investigate the effect of THF on CD73 activity, we assessed the ability of CD73 to converse AMP to Ado using HPLC. Fig. 1a was a representative HPLC chromatograph. In control group, the production of Ado by CD73 was 79.54 ± 3.69 nM/mg protein/10 min (Fig. 1b). However, the productions of Ado with the treatment of 5, 10 and 20 μM THF were significantly decreased by 47.37%, 68.95% and 85.67% (Fig. 1b), respectively. The production of Ado was also significantly decreased by APCP treatment but not by tylosin treatment (Fig. 1b). These data demonstrated that THF had a remarkable effect on decreasing the activity of CD73 in vitro.

### Cell proliferation decreased dramatically after treatment with THF

**Fig. 2:**
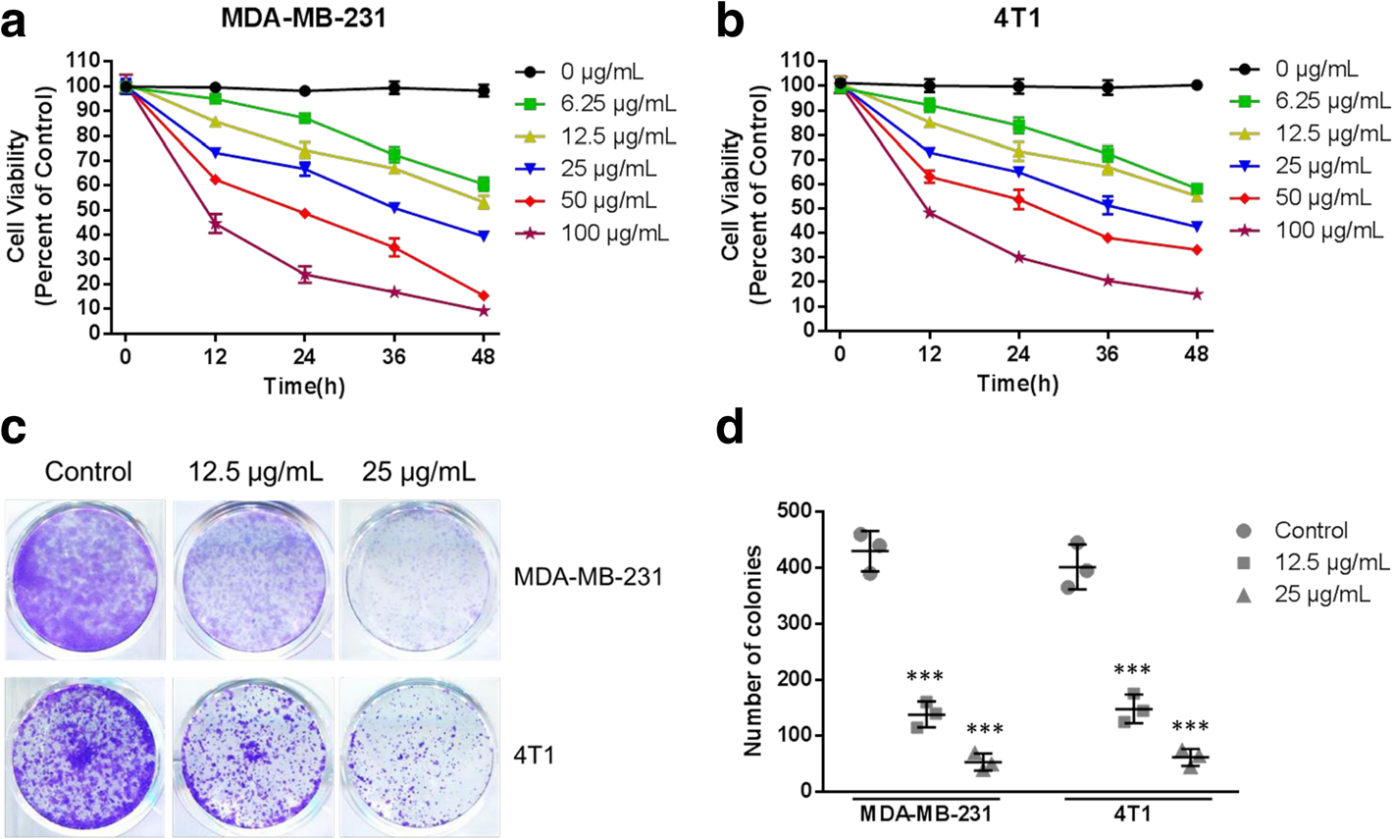
Cell viability decreased after treatment with THF. Cell viability was tested using Cell Counting Kit 8 at 0, 12,24,36,48 h after treatment with 0, 6.25, 12.5, 25, 50, 100 μg/mL THF in (**a**) MDA-MB-231 and (**b**) 4 T1 cells. Cell vitalities of both cells decreased with increasing dose or treatment period. **c** Representative colony formation of MDA-MB-231 and 4 T1 cells after treatment with solvent or 12.5, 25 μg/mL THF. THF significantly inhibited colony formation of both two cells (**d**). Data represent the mean ± S.D. of three independent experiments. (***P < 0.001 vs control)

To determine the effect and doses of THF, we detected the cell vitalities of MDA-MB-231 and 4 T1 cells using CCK-8. The results (Fig. 2a and b) showed that the cell vitalities of both cell lines decreased with increasing dose or treatment period. THF exhibited its effect in clear dose- and time-dependent manners. In the colony formation, the ability of cell colony was evaluated. MDA-MB-231 cells and 4 T1 cells were treated with THF, and then stained with crystal violet. Representative staining results were shown in Fig. 2c. With the treatment of 12.5 μg/mL THF, the number of colonies was reduced by 67.83% in MDA-MB-231 cells and 63.06% in 4 T1 cells (Fig. 2d). When the treatment doses increased to 25 μg/mL, the reduction reached to 87.59% in MDA-MB-231 cells and 83.41% in 4 T1 cells (Fig. 2d). Our data showed that THF dramatically inhibited colony formation of both cells.

### THF inhibited CD73 by down-regulating the activity instead of the expression of CD73

**Fig. 3:**
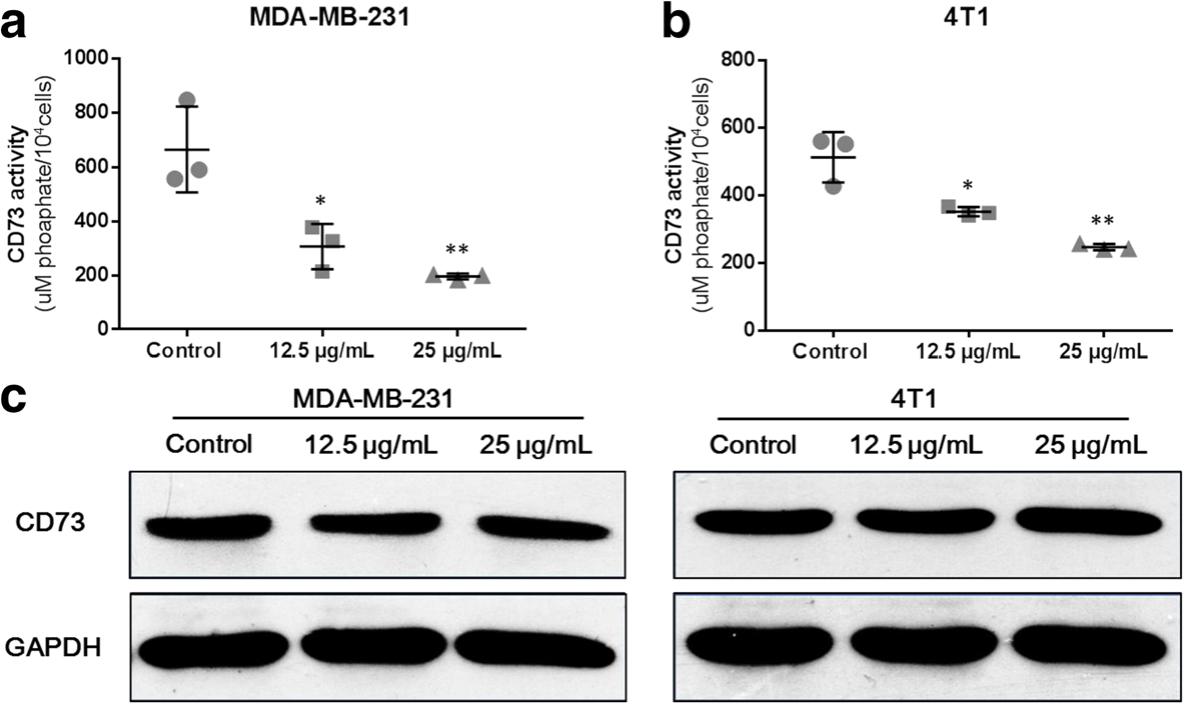
THF inhibited CD73 by down-regulating the activity instead of the expression of CD73. **a** and **b** Effect of THF on CD73 activity in breast cancer cells. After treatment with THF (12.5 or 25 μg/mL), CD73 activity significantly decreased in (**a**) MDA-MB-231 and (**b**) 4 T1 cells. **c** and **d** Western Blotting showing the expression of CD73 after 36 h treatment with solvent or 12.5, 25 μg/mL THF. Similar results were obtained from independent experiments in (**c**) MDA-MB-231 and (**d**) 4 T1 cells. Data represent the mean ± S.D. of three independent experiments. (*P < 0.05, **P < 0.01 vs control)

To further explore the mechanisms involved in the effect of THF on CD73, we measured the activity and expression of CD73 in MDA-MB-231 and 4 T1 cells. Compared with the controls, THF treatment significantly decreased the activity of CD73 in both cells in a dose dependent manner (Fig. 3a and b). At the concentration of 12.5 μg/mL, THF inhibited the activity of CD73 by 53.89% in MDA-MB-231 cells (Fig. 3a) and 31.41% in 4 T1 (Fig. 3b). Increased the concentration to 25 μg/mL, the inhibition rate reached to 70.53% in MDA-MB-231 cells (Fig. 3a) and 51.89% in 4 T1 (Fig. 3b). Similar inhibitions were achieved by APCP treatment but not by tylosin treatment in both cell lines (Additional file 1: Figure S1). Next, western-blot was used to determine the effect of THF on CD73 expression in MDA-MB-231 and 4 T1 cells. As shown in Fig. 3c and d, THF almost didn’t affect the expression of CD73 in both of these two cells. Together, the results above suggested that THF primarily exerted its effect by down-regulating the activity instead of the expression of CD73.

### THF suppressed cell migration and invasion via CD73 inhibition

**Fig. 4:**
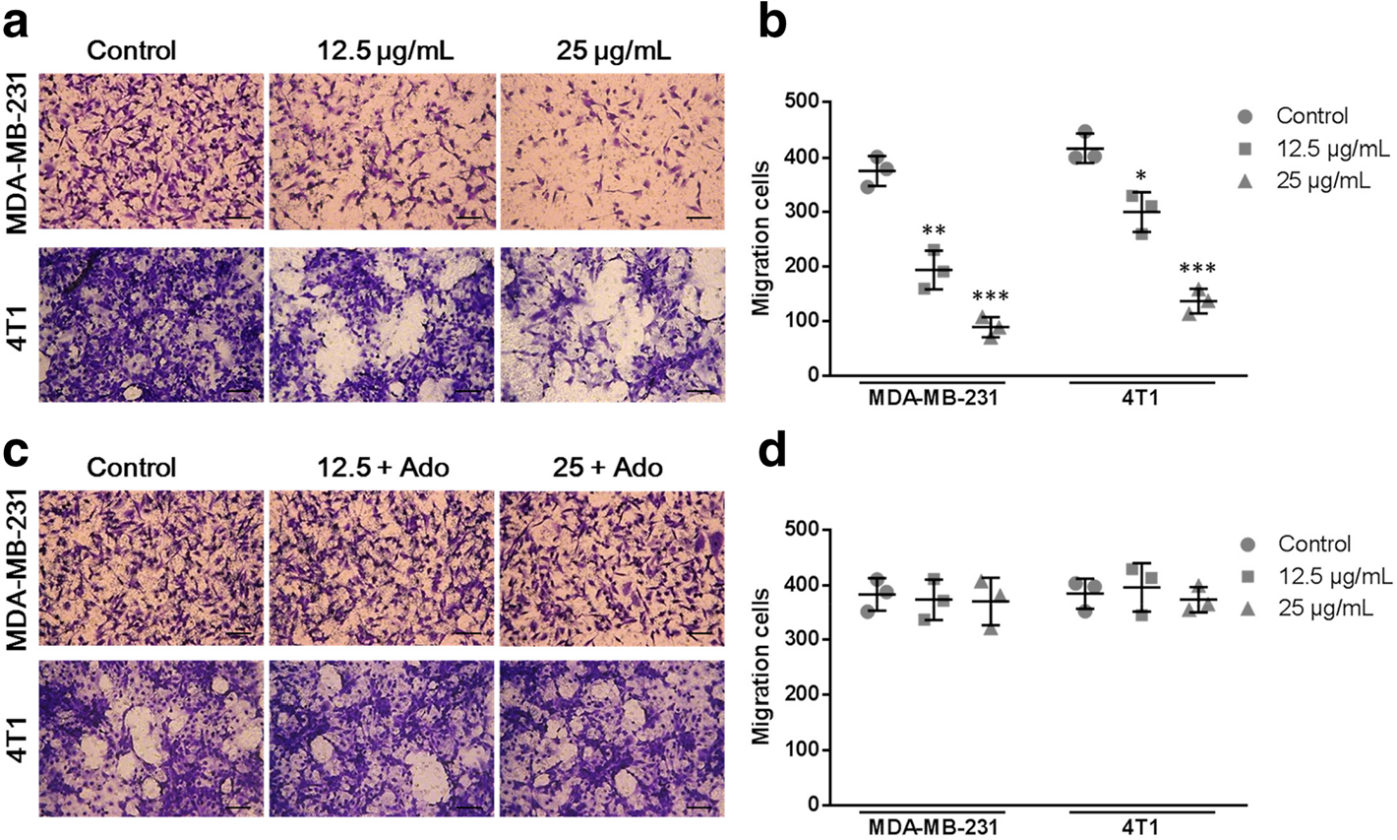
THF inhibited cell migration by decreasing CD73 activity. **a** Cells were cultured with solvent or 12.5, 25 μg/mL THF onto the upper well. After 36 h treatment, cells passed through the membrane into the lower well were stained and counted (Scale bar =25 μm). **b** Analysis of cell migration between control and THF treatment groups corresponding to the images in **a**. **c** In parallel experiments, exogenous Ado was added in THF treatment groups. After 36 h treatment, cells passed through the membrane into the lower well were stained and counted (Scale bar =25 μm). **d** Analysis of cell migration between control and treatment groups corresponding to the images in **c**. Data represent the mean ± S.D. of three independent experiments. (*P < 0.05, **P < 0.01, ***P < 0.001 vs control)

Ado produced by CD73 promotes cell migration and invasion, which are central in cancer metastatic process. In the migration assay, THF treatment caused a significant decrease in the amount of migrated cells. THF showed clear dose dependency in both MDA-MB-231 and 4 T1 cells (Fig. 4a). At the concentration of 12.5 μg/mL, THF inhibited cell migration by 48.31% in MDA-MB-231 and 27.86% in 4 T1 (Fig. 4b). When the concentration increased to 25 μg/mL, the inhibition rate reached to 76.20% in MDA-MB-231 and 67.01% in 4 T1 (Fig. 4b). However, the inhibition was rescued by adding Ado in both cells (Fig. 4c). Compared to the THF alone treatment group, the treatment of Ado (50 μg/mL) significantly increased the number of migrated cells in both cell lines. And, THF didn’t significantly decrease cell migration in Ado treatment groups compared to the control (Fig. 4d) (*P* > 0.05).

**Fig. 5:**
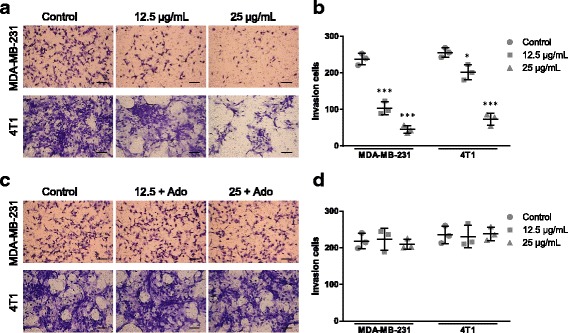
THF inhibited cell invasion by decreasing CD73 activity. **a** Cells were cultured with solvent or with 12.5, 25 μg/mL THF onto the upper well. After 36 h treatment, cells passed through the Matrigel into the lower well were stained and counted (Scale bar =25 μm). **b** Analysis of cell invasion between control and THF treatment groups corresponding to the images in **a**. **c** In parallel experiments, exogenous Ado was added in THF treatment groups. After 36 h treatment, cells passed through the Matrigel into the lower well were stained and counted (Scale bar =25 μm). **d** Analysis of cell migration between control and treatment groups corresponding to the images in **c**. Data represent the mean ± S.D. of three independent experiments. (*P < 0.05, **P < 0.01, ***P < 0.001 vs control)

Next, cell invasion was tested in MDA-MB-231 and 4 T1 cells (Fig. 5a and c). Similar to the results in the migration assay, THF treatment significantly decreased cell invasion in both cells (Fig. 5b), while THF didn’t cause significant decreases of cell invasion in Ado treatment groups compared to the control (Fig. 5d). Together, these results suggested that THF inhibited cell migration and invasion through down-regulation of CD73 activity.

### THF inhibited syngeneic tumor in vivo

**Fig. 6:**
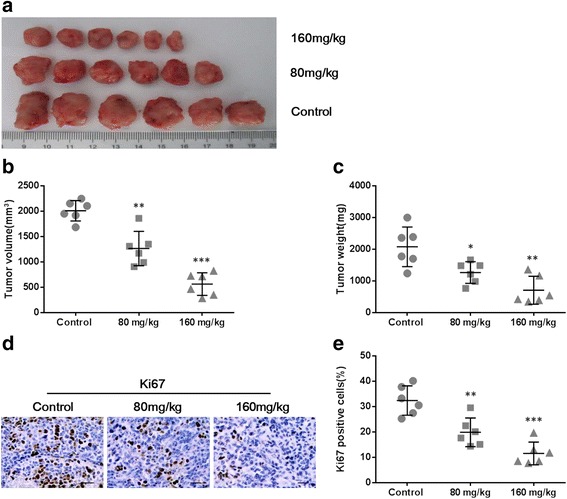
THF inhibited syngeneic tumor growth in vivo. Mice were injected s.c. with 1.5 × 10^6^ 4 T1 cells and then treated daily with THF at 80 or 160 mg/kg by gavage for 21 consecutive days. **a** The anatomic morphologic observation of syngeneic tumors in control and treatment groups. Tumor volume (**b**) and tumor weight (**c**) was calculated at indicated time points after treatment. **d** Representative Ki67 IHC staining of control or treated tumor tissues from mice (Scale bar =25 μm). **e** Quantitative analysis of Ki67 staining corresponding to the images in **d**. Data represent the mean ± S.D. of three independent experiments. (*P < 0.05, **P < 0.01, ***P < 0.001 vs control)

Since THF suppressed cell proliferation of cancer cell lines in vitro, we supposed it will further inhibit tumor growth in vivo. To confirm it, 4 T1 cells suspended in 100 μL PBS or PBS alone (normal control) were injected subcutaneously into female BABL/c mice to establish syngeneic tumors. Then the mice were treated once per day with 80 or 160 mg/kg THF by oral gavage. After 21-day treatment, all syngeneic tumors were collected (Fig. 6a). Quantitative analysis exhibited that the volume and weight of tumors in both treatment groups were significantly less than those in control group (Fig. 5b and c). Next, immunohistochemistry staining of Ki67 was performed in tumor tissues (Fig. 6d). The levels of Ki67 in treatment groups were significantly decreased compared to those in control (Fig. 6e). The data further confirmed that tumor growth was synergistically inhibited by THF.

### The antitumor effect of THF resulted in inhibition of metastasis and angiogenesis

**Fig. 7:**
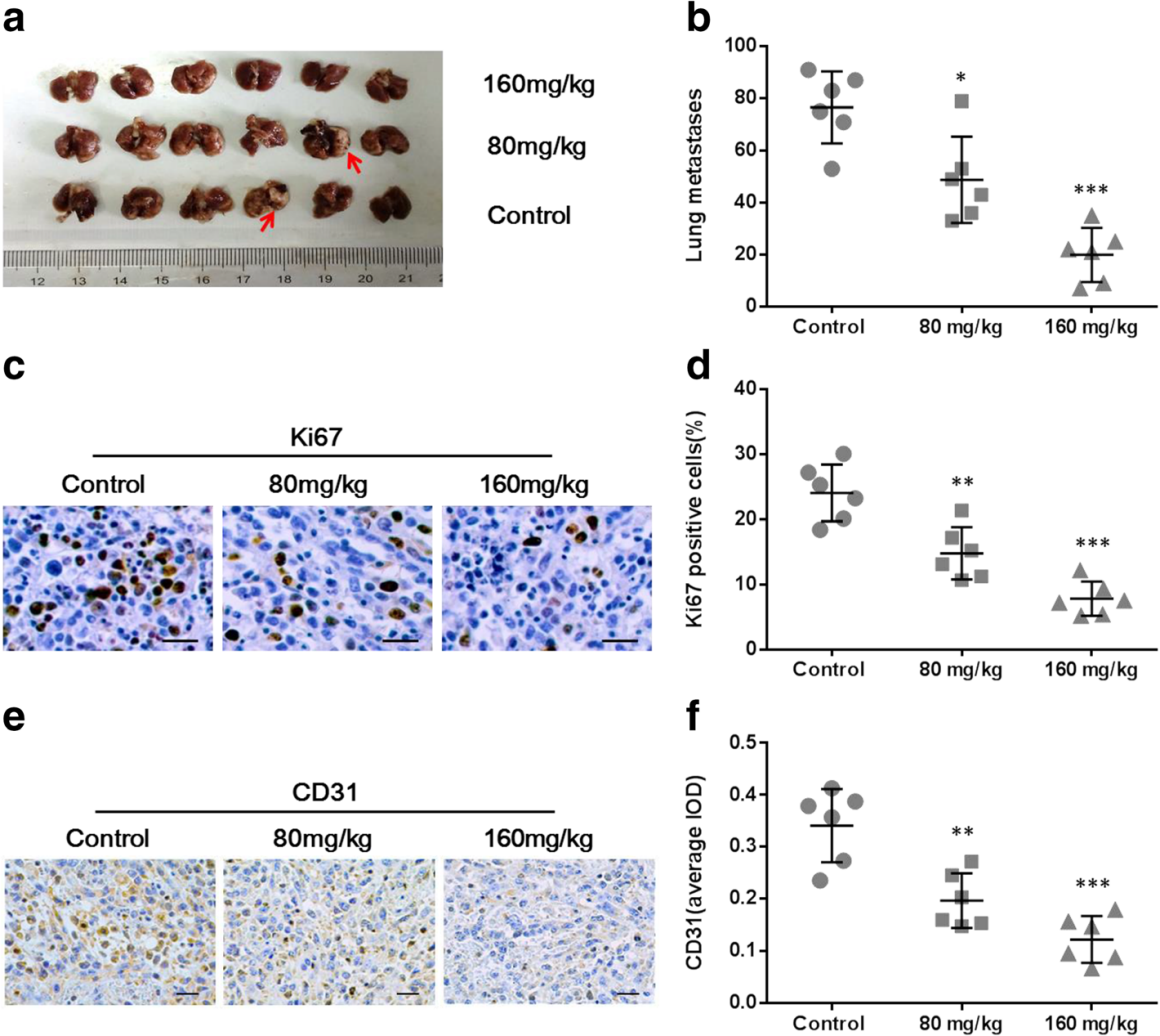
THF inhibited tumor metastasis via decreasing angiogenesis. **a** The complete lungs were fixed after the autopsy and the number of lung metastases foci (*arrow*) was counted. **b** Quantitative analysis of metastases corresponding to the images in (**a)**. **c** Representative Ki67 IHC staining of lung tissues from control or treated mice (Scale bar =25 μm). **d** Quantitative analysis of Ki67 staining corresponding to the images in (**c)**. **e** Representative CD31 IHC staining of control or treated tumor tissues from mice (Scale bar =25 μm). **f** Quantitative analysis of CD31 staining corresponding to the images in **e**. Data represent the mean ± S.D. of three independent experiments. (*P < 0.05, **P < 0.01, ***P < 0.001 vs control)

Unlike other mouse models of breast cancer, 4 T1 tumor can cause severe lung metastasis. As THF significantly inhibited the growth of primary tumors, we detected several indicators of pulmonary metastasis in all groups. Complete lungs were fixed after the autopsy and the number of metastatic foci (arrow) was counted (Fig. 7a). As show in Fig. 7b, the numbers of metastases in both low- and high-dosage treatment groups (48.83 ± 6.78 and 19.83 ± 4.26, respectively) were significantly less than those in the control group(76.67 ± 5.62). Besides, the IHC staining of Ki67 of lung tissues in both low- and high-dosage treatment groups (14.81 ± 1.65 and 7.83 ± 1.07, respectively) were significantly less than those in the control group(24.08 ± 1.78) (Fig. 7c and d). All these data demonstrated that THF inhibited pulmonary metastasis of 4 T1 tumor in vivo.

To further explore the mechanism involved in the inhibition of tumor by THF, we detected the expression of CD31 (a biomarker of angiogenesis) in primary tumor tissues by IHC staining (Fig. 7e). After semi quantitative analysis, the data showed that the level of CD31 in low- or high-dosage treatment groups significantly decreased compared to those in the control group (0.20 ± 0.02 or 0.12 ± 0.02 vs. 0.34 ± 0.03) (Fig. 7f). These suggested that the antitumor effect of THF via inhibition of CD73 also led to angiogenesis reducing.

## Discussion

CD73 is overexpressed in various types of cancer including breast cancer, gastric cancer, pancreatic cancer, colorectal cancer, prostate cancer and thyroid cancers [11, 19–25]. Increasing evidences have verified that CD73 plays a key role in cancer development, and CD73-derived Ado is also a crucial immunosuppressive factor, which promotes cancer progression including growth and metastasis [32, 33]. In breast cancer, CD73 overexpression was significantly associated with a worse prognosis, particularly in triple negative breast cancer (TNBC) [31]. Moreover, CD73 was deemed to be associated with resistance to antitumor agents. CD73 expression was revealed to be involved in an increased resistance to doxorubicin (DOX) in TNBC [31]. Particularly, due to the satisfactory effect on tumor-bearing mouse models, although there’s still a long way to go in clinical practice, anti-CD73 therapy has become a promising treatment for cancer patients in the future [34, 35].

In the present study, we found THF able to inhibit CD73 activity and then explored the effects of THF on breast cancer via this inhibition. We showed that THF inhibited breast tumor growth and metastasis, and this inhibition was mainly achieved by affecting the activity but not expression of CD73. So the effects of THF are similar to those of antibodies against CD73. However, THF, as a small molecular antibiotic, costs less and is easier to administrate (oral route) compared to antibody approaches (infusion or injection) [36]. Furthermore, small molecules have greater exposure within the tumor microenvironment owning to greater ability to cross physiological barriers, or the availability of diverse formulations that mitigate pharmacokinetic/pharmacodynamic challenges [36].

In this study, we firstly detected the effects of THF on CD73 using HPLC. We observed that THF inhibited the activity of CD73, which catalyzes AMP into Ado. Compared with that in control group, the level of Ado hydrolyzed by CD73 decreased significantly in THF treatment group. Furthermore, we tested the inhibition of CD73 by THF using Malachite Green Phosphate Assay Kit in breast cancer cell lines, including MDA-MB-231 and 4 T1, in both of which CD73 are highly expressed. Compared with that in control group, the catalytic activity of CD73 decreased dramatically after treatment with THF. Our data showed that THF inhibited the activity of CD73. It had been established that THF could reduce CD73 activity, while it was not clear whether THF affected the expression of CD73. To clarify this issue, we used western blot to detect the expression of CD73 in MDA-MB-231 and 4 T1 cells with treatment of THF. The results showed that THF had no significant effect on the expression of CD73 in both cell lines. THF was revealed to exert its effect by inhibiting the activity instead of the expression of CD73.

The ability to proliferate without limit is a major characteristic of malignant tumors, depending on the synthesis of a great deal of nucleotide, Ado included [37]. CD73 is a key enzyme in the generation of Ado, which also controls cell proliferation, angiogenesis and the immune response [3]. Overexpression of CD73 has been found to be associated with cancer growth. CD73 overexpression enhanced cell viability and promoted cell-cycle progression in pcDNA-NT5E transfected breast cancer cell [37]. The suppression of CD73 expression by shRNA could inhibit proliferation of breast cancer cell by inducing cell apoptosis and cell-cycle arrest [37]. Furthermore, treatment with APCP (α, β-methylene adenosine-5′-disphosphate), which is a specific competitive CD73 inhibitor, could suppress cancer cell proliferation in a dose-dependent manner [38]. In vivo, anti-CD73 mAb therapy significantly slowed 4 T1.2 and E0771 primary breast tumor growth in mice [11]. In tumorigenicity assay, tumor xenografts of the APCP treatment group showed delayed growth as their weight and volume were less than those in the control group [38]. In MB-MDA-231 cell, CD73 siRNA effectively inhibited CD73 gene expression at mRNA and protein level, resulting in growth suppression both in vivo and in vitro [39]. In our study, THF was found to inhibit CD73 activity and subsequently reduce the proliferation of breast cancer cell in vitro and the growth of tumor in vivo, which were similar to the results from previous reports. Taken together, these results support that CD73 promotes breast cancer growth, which can be retarded by CD73 inhibition.

Metastasis is the most vital attribute of malignant tumors and also is the ultimate cause of death in cancer patients [40, 41]. Migration and invasion of cancer cell are central in the metastatic process. Overexpression of CD73 promotes human breast cancer cell migration and invasion, which can be blocked by CD73 inhibitor [42]. In addition, CD73 siRNA effectively inhibits CD73 expression in breast cancer, leading to inhibition of invasion and migration [39]. In the present study, THF was shown to decrease the activity of CD73. We proposed that THF may have similar functions in inhibiting cell migration and invasion. In order to prove our hypothesis, we used transwell assay to test whether THF is capable of inhibiting the migration and invasion of MDA-MB-231 and 4 T1 cells via suppressing CD73. Our study showed that the migration and invasion of both two cells were reduced by THF in a dose-dependent manner, while this trend was reversed by the treatment of Ado. This suggested that THF inhibited cell migration and invasion by decreasing CD73 activity. In vivo, CD73 has been demonstrated to be associated with cancer metastasis in numerous studies, in which cancer metastasis was suppressed by CD73 inhibition. John Stagg etc. observed that anti-CD73 mAb therapy significantly reduced the numbers of spontaneous pulmonary metastases in breast cancer patients, even when primary tumors were of equivalent sizes [11]. In addition, CD73-deficient mice were guarded against lung metastasis of melanoma cells after intravenous injection [28]. In some studies, immunosuppressive action of CD73 promotes metastasis and CD73-derived Ado promotes melanoma metastasis by inhibiting natural killer cell cytotoxicity via A_2A_ receptors [33]. However, others argued that the prometastatic effect results from CD73 expression on endothelial cells and is independent from its immunosuppressive action [28]. The question whether the prometastatic effect of CD73 has an immune component need to be explored in future study. In this study, we found that THF as a CD73 inhibitor substantially delayed breast tumor growth and suppressed lung metastasis.

Furthermore, we explored the mechanisms of THF in tumor inhibition by detecting angiogenesis. The development of tumor requires an abundant supply of blood. In the progression and enlargement of solid neoplasms, an important factor is angiogenesis, which also has a close relation to invasion and metastasis [43]. CD73 promotes tumor angiogenesis, which had been established both in vivo and in vitro [44–46]. We detected the expression level of CD31, which can be used as a marker of density of microvessel [47], using IHC staining in both the control group and THF treatment groups. We found that THF considerably decreased the vascular density in 4 T1 breast tumor. We first demonstrated the antitumor effect of THF in breast cancer, and it is likely to be achieved by reducing angiogenesis via CD73 inhibition. In addition, CD73 has been found associated with resistance to antitumor agents [31, 48–50]. THF might be able to inhibit the drug resistance caused by CD73, although it needs to be identified in future studies.

## Conclusions

Our study not only revealed that THF significantly restricted breast tumor growth and metastasis, but also demonstrated that the inhibition effect of THF on CD73 activity may contribute to its anti-tumor effects. Our study suggested that THF might present a promising prospect for breast cancer therapy.

## Additional file

Additional file 1: Figure S1. The activity of CD73 was decreased by THF as well as APCP, but not by tylosin. **a** and **b** Effect of THF, APCP or tylosin on CD73 activity in breast cancer cells. CD73 activity was significantly decreased by THF or APCP, but not by tylosin in (**a**) MDA-MB-231 and (**b**) 4 T1 cells. Data represent the mean ± S.D. of three independent experiments. (**P* < 0.05, ***P* < 0.01, ****P* < 0.001 vs control). (PPTX 107 kb)

## Abbreviations

Ado: Adenosine
AMP: Adenosine monophosphate
APCP: α, β-Methylene adenosine-5′-disphosphate
CCK-8: Cell Counting Kit-8
DAB: 3, 3′-Diaminobenzidine
ECM: Extracellular matrix
FBS: Fetal bovine serum
HPLC: High performance liquid chromatography
IHC: Immunohistochemistry
IPP: Image-pro-plus
OD: Optical density
SD: Standard deviation
THF: Tiamulin hydrogen fumarate
TNBC: Triple negative breast cancer
