# Supplementary figures and images for: Tiamulin inhibits breast cancer growth and pulmonary metastasis by decreasing the activity of CD73
Source: BMC Cancer. 2017 Apr 11;17:255. doi: 10.1186/s12885-017-3250-4 (PMC5387263; doi:10.1186/s12885-017-3250-4)

## Slide 1
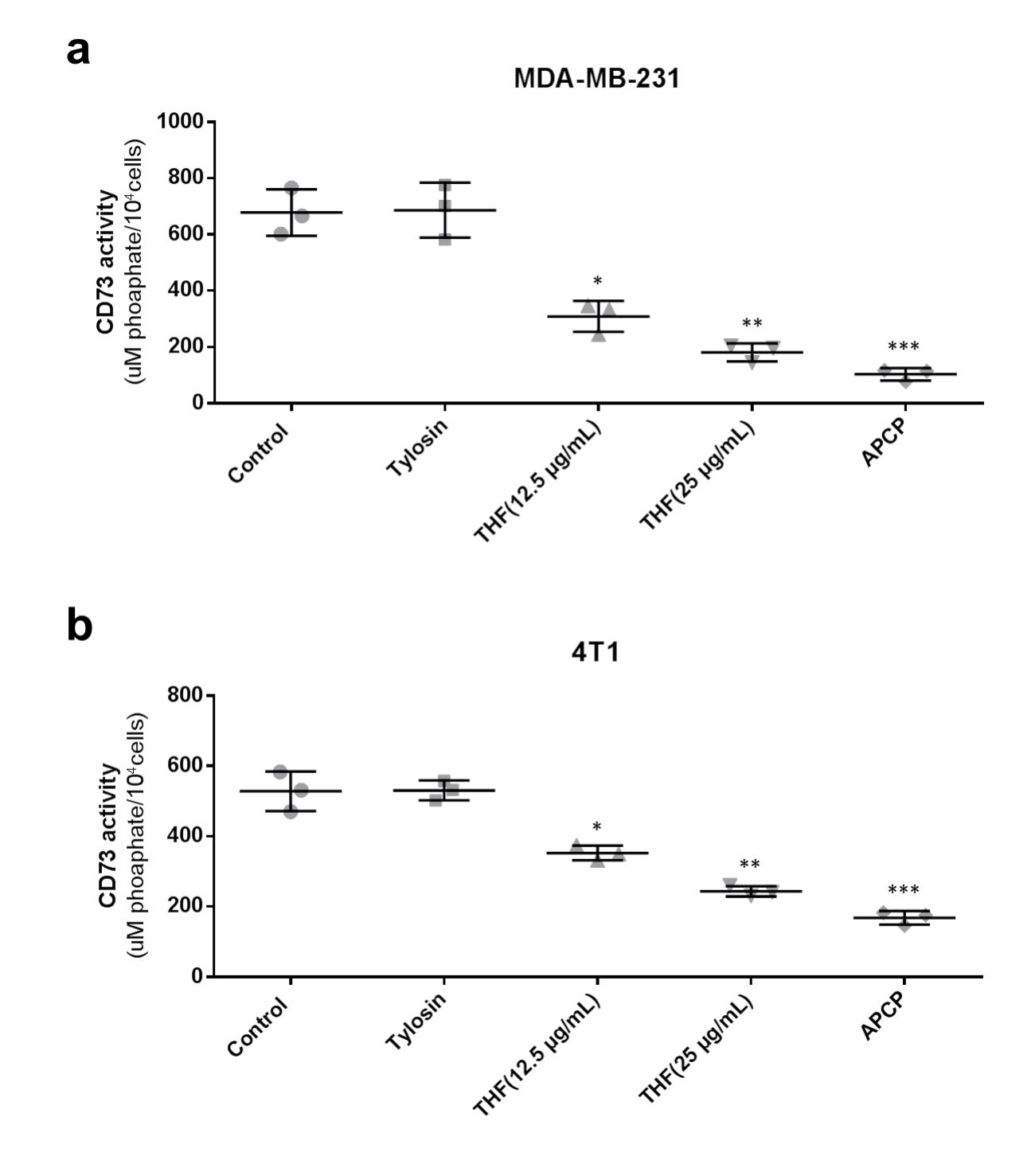

Supplement: Additional file 1: Figure S1. — The activity of CD73 was decreased by THF as well as APCP, but not by tylosin. a and b Effect of THF, APCP or tylosin on CD73 activity in breast cancer cells. CD73 activity was significantly decreased by THF or APCP, but not by tylosin in (a) MDA-MB-231 and (b) 4 T1 cells. Data represent the mean ± S.D. of three independent experiments. (*P < 0.05, **P < 0.01, ***P < 0.001 vs control). (PPTX 107 kb) [file 12885_2017_3250_MOESM1_ESM.pptx]
